# Co-existence of peripheral fatigue of the knee extensors and jump potentiation after an incremental running test to exhaustion in endurance trained male runners

**DOI:** 10.3389/fspor.2023.1267593

**Published:** 2023-11-09

**Authors:** Gonzalo Márquez, Jorge González-Hernandez, Pedro Jiménez-Reyes, David Colomer-Poveda, Daniel Boullosa

**Affiliations:** ^1^ Department of Physical Education and Sport, Faculty of Sports Sciences and Physical Education, University of A Coruna, A Coruña, Spain; ^2^ Faculty of Health Sciences, Universidad Europea de Canarias, Tenerife, Spain; ^3^ Center for Sport Studies, Rey Juan Carlos University, Madrid, Spain; ^4^ Faculty of Sports Sciences and Physical Activity, Universidad de León, León, Spain

**Keywords:** vertical jump, neuromuscular fatigue, twitch interpolation technique, post activation potentiation (PAP), incremental running test

## Abstract

The aim of the present study was to investigate the effect of an incremental running exercise until exhaustion on twitch responses and jump capacity in endurance trained runners. For this purpose, 8 experienced endurance male runners were required to perform neuromuscular function tests before and after a submaximal running bout (control condition -CTR-) or an incremental running test to volitional exhaustion (experimental conditions -EXP-). The twitch interpolation technique was used to assess voluntary activation and muscle contractile properties before and after each condition (CTR and EXP). Countermovement jump was also used to assess the stretch-shortening cycle function before and after both conditions. In addition, rating of perceived exertion, heart rate, blood lactate and skin temperature were also recorded. Only EXP improved jump performance, however, it was also accompanied by a reduction in maximal voluntary contraction and the peak twitch force of the knee extensors evoked by electrical stimulation at 10 Hz (Db10). It is likely that reductions in maximal voluntary contraction may be related to an excitation-contraction coupling failure (i.e. low-frequency fatigue) as suggest the reduction in the Db10. The current results confirm that acute changes in jump performance may not be appropriate to evaluate acute fatigue in endurance trained runners.

## Introduction

Post-activation performance enhancement (PAPE) refers to the performance enhancement in a specific test or exercise following the positive acute effect of a voluntary conditioning activity (CA). This term has served to differentiate between muscle performance enhancement measured with the twitch interpolation technique (TIT) after a voluntary activity (i.e., post-activation potentiation; PAP), and performance enhancement measured in sport settings (i.e., PAPE) (1, 2). While Blazevich and Babault (1) have suggested that the timing and the mechanisms may differ between PAPE and PAP, some evidence suggest their co-existence (3–5), therefore the “gold standard” criterium for differentiating between PAPE and PAP should be the test used for its verification (2). In practice, power trained athletes expect to increase their muscle power a few minutes after a high-intensity resistance exercise or a plyometric exercise (6). Meanwhile, PAPE responses have also been reported in endurance trained athletes (6). However, while endurance athletes can also exhibit enhanced muscle power after a high-intensity exercise or a plyometric exercise (7, 8), it seems that they also can exhibit PAPE after specific endurance exercises (9). For instance, endurance trained runners have reported enhanced jump capacity after different incremental (10), interval (11) and continuous running exercises (12), despite evidence of metabolic, muscle or perceptual fatigue.

Classically, it is assumed the co-existence of both fatigue and potentiation mechanisms after a CA (13). There is a window of opportunity after completion of a CA in which muscle potentiation mechanisms are still present while the acute fatigue is reduced, thus resulting in a positive potentiation/fatigue balance (13). Previously, Boullosa et al. (14) observed an enhancement in jump capacity after an incremental test in the field in which the endurance runners also exhibited a reduction of the peak force (F_peak_) at the end of the eccentric phase of a countermovement jump (CMJ). Furthermore, those athletes with the lower F_peak_ loss were those who increased more the peak power output (PO_peak_) and therefore the height of the CMJ (H_CMJ_). However, the authors (14) did not verify the origin and levels of fatigue with the twitch interpolation technique (TIT) to effectively verify if the force-time (F-t) recordings during the CMJ were, in fact, related to neuromuscular fatigue responses. The TIT is a classical method of neuromuscular assessment, first described by Merton (15), which is often used in research settings to study central and peripheral mechanisms of fatigue by means of recording the evoked forces produced by electrical supramaximal nerve or muscle stimulation of a specific muscle group (16).

Thus, the purpose of the current brief research report was to verify the relationships of the twitch responses and jump performance in endurance trained runners after an incremental running test until exhaustion. Based on the previous evidence, we hypothesized that the reduction in the eccentric force during the CMJ would be related to the fatigue detected with the TIT, despite the observation of post-running jump potentiation.

## Material and methods

### Participants

Eight well trained runners [age: 28.1 ± 8.18 years, height: 175 ± 7.13 cm, body mass: 69.39 ± 3.94 kg; maximal aerobic speed (MAS): 18.9 ± 0.9 km·h^−1^], with at least ten years of experience in endurance training, were recruited and gave their written informed consent in agreement with the last version of the Declaration of Helsinki. None of the participants reported any history of orthopaedic injuries, neuromuscular or cardiovascular disorders that could affect the results of the study. Participants were also required to refrain from consuming caffeinated or alcoholic drinks or exercise 48 h before the study. The study was approved by the Catholic University of Murcia (UCAM) Ethics Committee (reference number: 171114).

### Study design

**Figure 1:**
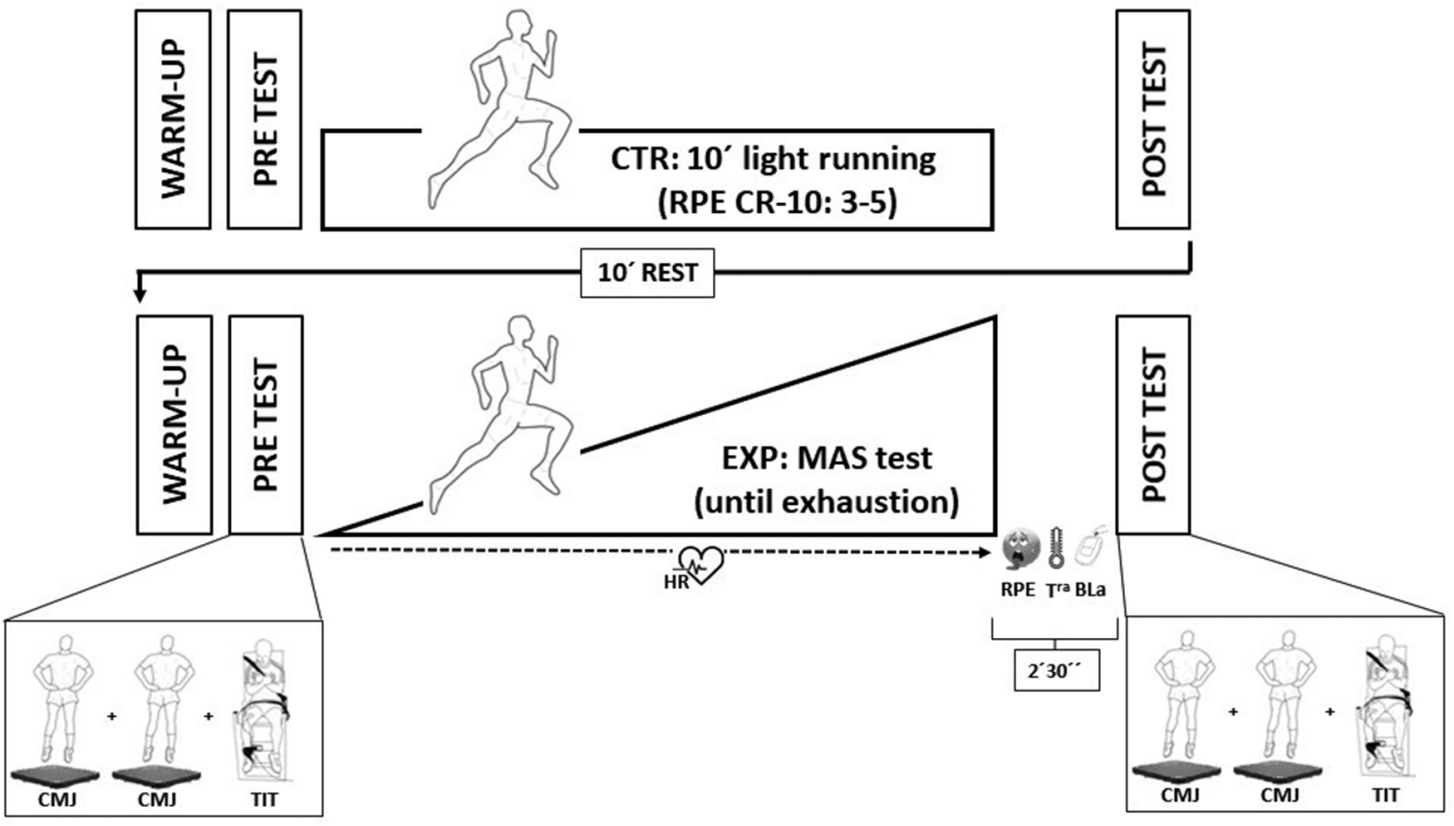
Overview of the experimental procedures. Please note that between the end of the running bouts and the first jump performed at 2'30", RPE, skin temperature and BLa were recorded. Then, the TIT evaluation was performed between 3'30" and 4' post-running. These time points were the same for all the participants in both experimental conditions.

A repeated measures design was implemented to compare the acute mechanical, neuromuscular, and metabolic responses after performing two different running protocols. For this purpose, each participant undertook one experimental session that consisted of the control (CTR) and the experimental (EXP) evaluations performed in that order. For the CTR condition, participants were required to perform neuromuscular function tests before and after a submaximal running exercise. Then, after 10 min of rest, for the EXP condition, participants repeated the neuromuscular function tests before and after an incremental running test until volitional exhaustion. The neuromuscular assessments performed before and after each condition consisted in a vertical CMJ test and recording of the evoked responses induced by electrical stimulation of the femoral nerve during a maximal voluntary contraction (MVC) and at rest (i.e., superimposed and potentiated twitches). Figure 1 depicts an overview of the general experimental design.

The twitch interpolation technique (TIT) was used to assess voluntary activation and muscle contractile properties of the knee extensors before (PRE) and after (POST) each condition (CTR and EXP). Countermovement jump (CMJ) was also used to assess the stretch-shortening cycle function before and after both conditions. In addition, rating of perceived exertion (RPE), heart rate (HR), blood lactate [La] and skin temperature were also recorded. All the measurements were made in a room inside the athletic stadium, with a thermoneutral room temperature during the whole experiment (temperature: 20–22°C; relative humidity: 52%–54%). Subjects were not previously familiarized with the strength tests used for this study in a separate session because they routinely performed CMJs and different strength tests (e.g., squats and knee extensions) as a part of their training monitoring. For the TIT, they were specifically familiarized in the same session with one trial during the warm-up in both conditions (CTR and EXP).

### Procedures

#### Countermovement jump test

Participants were instructed to start in an upright position, and then to jump “as high as possible”. The hands were akimbo throughout the test attempts in order to eliminate the effect of arm swing during jumping. During the squat phase of the movement, the angular displacement of the knee was standardized so that participants were required to bend their knees to approximately 90°. A 90° knee bend was merely a reference value and not an inclusion criterion (17, 18).

Vertical ground reaction forces were recorded with a force plate (Kistler 9290AD; Kistler Inc. Switzerland) to compute vertical acceleration of the centre of mass (CoM) at 500 Hz. Then, vertical displacement of the CoM was computed using the double integration method (19). The jump height was defined as the difference between the height of the COM in the standing position and the apex (20). Vertical stiffness (K_vert_) during the CMJ was defined as: F_peak_/*Δ*L; where F_peak_ is the peak vertical ground reaction forces and *Δ*L is the vertical displacement of the CoM from the starting position to the lowest position (14, 17, 18).

#### Neuromuscular function assessment

Neuromuscular function was tested before and after each condition (CTR and EXP). For this purpose, participants were seated in a custom-made chair with both knees flexed at 90° and the torso restrained with belts to avoid any displacement. Right leg was strapped to a force transducer (NL63-200 Kg; Digitimer, Welwyn Garden City, United Kingdom) just above the malleoli. Transcutaneous electrical muscle stimulation (200 *μ*s) was applied to the right knee extensors using a constant-current stimulator (DS7AH, Digitimer Ltd, Welwyn Garden City, Hertfordshire, UK). Rectangular (5 × 10 cm) self-adhesive surface electrodes (Compex Inc., Switzerland) were placed proximally (over the upper third of the muscle) and distally (just above the patella) over the knee extensors. Single stimuli were delivered to the relaxed muscle beginning at 100 mA and increasing by 20 mA until a plateau occurred in twitch amplitude. Supramaximal stimulation was ensured by increasing the final intensity by 30% (mean current of 313 ± 27 mA).

Participants were asked to perform a knee extensor MVC of ∼3 s before and after each running condition (CTR and EXP). During each MVC, a high-frequency doublet (100 Hz) was superimposed at 1.5 s. In addition, a 100 Hz doublet, a 10 Hz doublet, and a single twitch (Tw) were delivered at rest after each MVC.

Global fatigue was determined by measuring the peak MVC recorded before the superimposed doublet at 100 Hz (Db100_sup_). The indices of peripheral fatigue were determined by the mechanical responses to single stimulations (potentiated peak twitch, Tw), potentiated high- and low- frequency doublets (Db100 and Db10, respectively), and the ratio of paired stimulation peak forces at 10 Hz over 100 Hz (10:100 Ratio).

Central fatigue was evaluated by measuring voluntary activation (VA). This parameter was calculated from the maximal force attained during the MVC (F_max_), the force before the superimposed doublet (F_before_), the peak force following the superimposed doublet (Db100_sup_), and Db100 (21): VA = [1−[(Db100_sup_−F_before_) × (F_before_ / F_max_)]/Db100] × 100.

#### Blood lactate, RPE, HR and skin temperature

Mean and maximal HR were measured during both conditions using a HR monitor (V800, Polar Electro Oy, Finland). Rating of perceived exertion (RPE) was assessed using the modified Borg CR-10 scale (22). Blood lactate (BLa) concentration was determined using a portable lactate analyser (Lactate Pro, Arkray, Japan) from capillary blood samples drawn from the fingertip after each running bout. Skin temperature was recorded as a surrogate of muscle temperature from a non-contact commercial infrared thermometer (Model FTN_B, Medisana; Germany) previously validated (23). We have assessed these physiological variables (BLa and skin temperature) along with HR and RPE, to better characterize the exercise loading imposed in the EXP and CTR conditions.

#### Running exercises

The study comprised two different running protocols within the same session. During CTR, participants were asked to run during 10 min at their self-selected pace that corresponded to 5 points in the CR-10 scale (participants usually included a CR scale to rate intensity during their trainings). In EXP, participants completed an incremental maximal running track test until volitional exhaustion. The protocol was implemented using the VAMRAM software (Ergotech, Barcelona, Spain) which imposed an increased speed that started at 2.22 m·s^−1^ and that was progressively increased by 0.28 m·s^−1^ every min until volitional exhaustion. The MAS was considered as the highest velocity reached in the last completed stage (24). The incremental running exercise was selected because it has been the most common evaluation of jump potentiation in endurance athletes; it induces fatigue of different origins as a consequence of running until exhaustion; and allows a valid characterisation of athletes with the MAS recorded. The duration of the CTR protocol was based on the estimation of the duration of the EXP protocol (estimations done based on the participants self-reported MAS).

### Statistical analyses

Statistical analysis was performed using a commercially available software (SPSS 23.0, IBM Corp. IBM SPSS Statistics for Windows, Armonk, NY, USA). Data are presented as group mean values ± standard deviation (SD). Reliability was determined for dependent variables using coefficient of variation (CV = SD/Mean × 100) and intra-class correlation coefficients (ICCs) [single measurement, absolute agreement, 2-way-mixed-effects model (3,1)] of the PRE values corresponding to each condition. The ICC was interpreted with values below 0.5 indicating low reliability, values between 0.5 and 0.75 indicating moderate reliability, values between 0.75 and 0.9 indicating good reliability, and values higher than 0.90 indicating excellent reliability. Then, the Shapiro-Wilk analysis was used to test the normal distribution of the data. All the variables were normally distributed, except RPE, which was log transformed prior to further statistical testing.

A two-way repeated measures analysis of variance (RM-ANOVA) with condition (CTR and EXP) and time (Pre- and Post-) as factors was performed for the following dependent variables: MVC, VA(%), Db100, Db10, 10:100 Ratio, Tw, H_CMJ_, F_peak_, *Δ*L and K_vert_. For the RM-ANOVA calculations, the assumption of Sphericity was tested with the Mauchly's test. In cases where the assumption of Sphericity was violated, the Greenhouse-Geisser correction was applied. In case of significant *F*-values (*p* ≤ 0.05), post-hoc analyses were performed using paired comparisons with Bonferroni correction. Partial Eta Square (*η*_p_^2^) was used for effect-size calculation, with values ≥0.01, ≥0.06, ≥0.14 indicating small, moderate, and large effects, respectively.

In order to compare cardiovascular (HR_mean_ and HR_max_), metabolic (BLa) and perceptual (RPE) responses during each running condition (CTR vs. EXP) paired student *t*-test were used. Furthermore, to test the relationships between the changes (DELTA from PRE to POST values in %) of the previously mentioned dependent variables we have used the Pearson product correlation coefficient (r).

## Results

### Reliability

Intra-class correlation coefficients for selected mechanical, physiological and neuromuscular parameters ranged from 0.57 to 0.99 (see Supplementary Table S1 for detailed CV and ICC values for each variable). The reliability analysis showed good to excellent reliability (ICC >0.75) for all selected variables, except for the VA and K_vert,_ which showed moderate reliability (0.57 and 0.58, respectively).

### Blood lactate, RPE, HR and skin temperature

**Figure 2:**
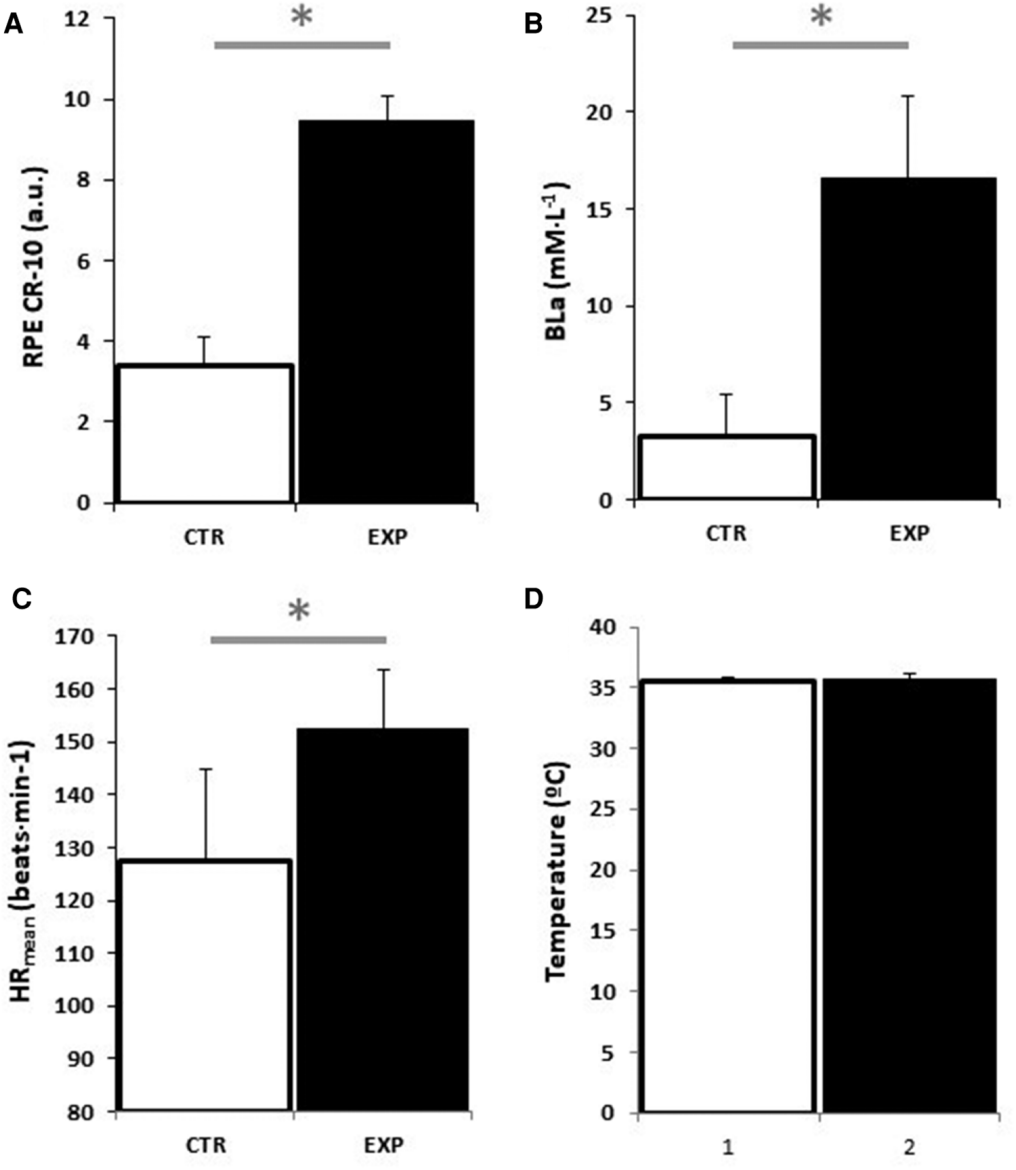
The figure 2 depicts now physiological and perceptual data obtained during and after the EXP (black) and CTR (white) conditions for RPE (**A**), BLa (**B**), hR_mean_ (**C**) and skin temperature (**D**).

The physiological and perceptual data obtained from the MAS test (HR_max_: 181 beats·min^−1^; BLa: 16.6 mM·L^−1^; and RPE: 9.4 a.u. of Borg's CR-10 scale) confirmed the maximal effort in EXP. In contrast, the data from the CTR condition confirmed the submaximal effort (HR_max_: 157 beats·min^−1^; Bla: 3.3 mM·L^−1^; and RPE: 3.4 a.u. of Borg's CR-10 scale). However, skin temperature was similar between conditions (*p* > 0.05). Physiological and perceptual data are depicted in Figure 2.

### Countermovement jump assessment

The data of the selected CMJ variables are depicted in Table 1. H_CMJ_ and PO_peak_ were higher after completing the MAS test (4.9 and 7.0%, respectively; *p* ≤ 0.05) than after CTR (−0.2 and −1.5%, respectively). However, F_peak_ decreased after both conditions (CTR: −3.3%; EXP: −4.8%; *p* ≤ 0.05). *Δ*L and K_vert_ did not change significantly in both conditions (*p* > 0.05).

**Table 1:** Data of the selected CMJ variables obtained before (PRE) and after (POST) both experimental conditions (CTR and EXP).

Variable	Condition	PRE	POST	DELTA (%)	Condition	Time	Condition*time
H_CMJ_ (cm)	CTR	40.7 ± 5.2	40.6 ± 5.3	−0.2 ± 4.2	F_(1, 7) _= 8.919; *p*_ _= 0.020; *η*_p_^2^_ _= 0.560	F_(1, 7) _= 3.941; *p*_ _= 0.088; *η*_p_^2^_ _= 0.360	F_(1, 7) _= 6.278; *p* = 0.041; *η*_p_^2 ^= 0.473
	EXP	41.2 ± 5.4	43.1 ± 4.5*^,^^#^	4.9 ± 4.9			
*Δ*L (cm)	CTR	31.2 ± 7.5	31.8 ± 5.1	5.2 ± 20.1	F_(1, 7) _= 0.730; *p*_ _= 0.421; *η*_p_^2^_ _= 0.094	F_(1, 7) _= 3.160; *p*_ _= 0.119; *η*_p_^2^_ _= 0.311	F_(1, 7) _= 1.921; *p* = 0.208; η_p_^2 ^= 0.215
	EXP	30.8 ± 7.0	34.3 ± 6.6	12.9 ± 15.4			
F_peak_ (kN)	CTR	1.73 ± 0.27	1.65 ± 0.29*	−4.8 ± 6.0	F_(1, 7) _= 0.087; *p*_ _= 0.777; *η*_p_^2^_ _= 0.012	F_(1, 7) _= 13.662; *p*_ _= 0.008; *η*_p_^2^_ _= 0.661	F_(1, 7) _= 0.248; *p* = 0.634; *η*_p_^2 ^= 0.034
	EXP	1.71 ± 0.30	1.66 ± 0.35*	−3.3 ± 5.6			
K_vert_ (kN·cm^−1^)	CTR	5.4 ± 1.0	5.3 ± 1.0	−1.0 ± 11.6	F_(1, 7) _= 0.061; *p*_ _= 0.811; η_p_^2^_ _= 0.009	F_(1, 7) _= 4.404; *p*_ _= 0.074; *η*_p_^2^_ _= 0.386	F_(1, 7) _= 4.124; *p* = 0.082; *η*_p_^2 ^= 0.371
	EXP	5.8 ± 1.7	5.0 ± 1.5	−13.0 ± 12.7			
PO_peak_ (W·BW^−1^)	CTR	48.9 ± 5.9	48.1 ± 4.9	−1.5 ± 4.6	F_(1, 7) _= 6.892; *p*_ _= 0.034; *η*_p_^2^_ _= 0.496	F_(1, 7) _= 3.690; *p*_ _= 0.096; *η*_p_^2^_ _= 0.345	F_(1, 7) _= 8.839; *p* = 0.021; *η*_p_^2 ^= 0.558
	EXP	48.7 ± 5.1	51.9 ± 4.8*^,^^#^	7.0 ± 6.7			

H_CMJ_, Countermovement jump height; *Δ*L, vertical displacement of the center of mass during the unloading phase of the CMJ; F_peak_, peak force during the countermovement jump; K_vert_, vertical stiffness; PO_peak_, relative peak power during the push off phase of the countermovement jump; CTR, control condition; EXP, experimental condition.

*Differences between PRE & POST.

^#^Differences between conditions (CTR vs. EXP).

### Neuromuscular assessment

The data of the selected neuromuscular variables are depicted in Table 2. Isometric MVC was reduced after EXP (−11.9%; *p* ≤ 0.05). Furthermore, a reduction in the Db10 (−14%; *p* ≤ 0.05) and the Ratio 10:100 (−18%; *p* ≤ 0.05), without changes in the Db100 and VA (%) was also observed in EXP. In contrast, all the variables remained unchanged after the CTR condition (see Table 2).

**Table 2:** Data of the selected neuromuscular variables obtained from the TIT measurements before (PRE) and after (POST) both experimental conditions (CTR and EXP).

Variable	Condition	PRE	POST	DELTA (%)	Condition	Time	Condition*time
MVC (*N*)	CTR	521 ± 127	531 ± 99	4 ± 14	F_(1, 7) _= 1.059; *p*_ _= 0.338; *η*_p_^2^_ _= 0.131	F_(1, 7) _= 2.033; *p*_ _= 0.197; *η*_p_^2 ^= 0.225	F_(1, 7) _= 46.670; *p*_ _= 0.000; *η*_p_^2^_ _= 0.870
	EXP	536 ± 113	474 ± 102*^,^^#^	−11 ± 9			
VA (%)	CTR	93 ± 8	90 ± 8	−2 ± 9	F_(1, 7) _= 3.334; *p*_ _= 0.111; *η*_p_^2^_ _= 0.323	F_(1, 7) _= 1.569; *p*_ _= 0.251; *η*_p_^2^_ _= 0.550	F_(1, 7) _= 0.394; *p*_ _= 0.550; *η*_p_^2^_ _= 0.053
	EXP	89 ± 8	85 ± 12	−5 ± 11			
Ratio 10:100	CTR	0.87 ± 0.1	0.84 ± 0.1	−1 ± 15	F_(1, 7) _= 0.631; *p*_ _= 0.453; *η*_p_^2^_ _= 0.083	F_(1, 7) _= 8.210; *p*_ _= 0.024; *η*_p_^2^_ _= 0.540	F_(1, 7) _= 15.503; *p*_ _= 0.006; *η*_p_^2^_ _= 0.689
	EXP	0.91 ± 0.2	0.75 ± 0.1*^,^^#^	−18 ± 6			
Db100 (*N*)	CTR	233 ± 40	235 ± 32	2 ± 14	F_(1, 7) _= 0.010; *p*_ _= 0.924; *η*_p_^2^_ _= 0.001	F_(1, 7) _= 0.277; *p*_ _= 0.615; *η*_p_^2^_ _= 0.038	F_(1, 7) _= 0.314; *p*_ _= 0.593; *η*_p_^2^_ _= 0.043
	EXP	231 ± 49	238 ± 32	5 ± 15			
Db10 (*N*)	CTR	200 ± 34	198 ± 36	0 ± 10	F_(1, 7) _= 4.998; *p*_ _= 0.060; *η*_p_^2^_ _= 0.417	F_(1, 7) _= 20.652; *p*_ _= 0.003; *η*_p_^2^_ _= 0.747	F_(1, 7) _= 5.786; *p*_ _= 0.047; *η*_p_^2^_ _= 0.453
	EXP	206 ± 37	176 ± 27*^,^^#^	−14 ± 8			
Tw (*N*)	CTR	161 ± 23	160 ± 24	−1 ± 4	F_(1, 7) _= 2.136; *p*_ _= 0.187; *η*_p_^2^_ _= 0.234	F_(1, 7) _= 4.085; *p*_ _= 0.083; *η*_p_^2^_ _= 0.369	F_(1, 7) _= 3.348; *p*_ _= 0.110; *η*_p_^2^_ _= 0.324
	EXP	164 ± 32	149 ± 25	−8 ± 10			

MVC, maximal voluntary contraction; VA, voluntary activation; Ratio 10:100, ratio of doublets at 10 and 100 Hz; Db100, doublet at 100 Hz; Db10, doublet at 10 Hz; Tw, Single twitch; CTR, control condition; EXP, experimental condition.

* Differences between PRE & POST.

^#^ Differences between conditions (CTR vs. EXP).

Finally, significant correlations were observed between the changes (Δ) in K_vert_ and H_CMJ_ (*r* = −0.747; *p* = 0.001), and between the changes (Δ) in MVC and the ratio 10:100 (*r* = 0.569; *p* = 0.022).

## Discussion

We determined the effect of an incremental running exercise until exhaustion on peripheral and central components of knee extensors fatigue, and their association with jump capacity in endurance trained runners. Our results confirmed the expected improvement in jump performance after an incremental running test as previously described (14). The post-incremental running jump potentiation occurred despite the reduction in the isometric peak force of the knee extensors. The reduction in MVC was possibly due to an excitation-contraction coupling failure (i.e., low-frequency fatigue). However, the reductions in F_peak_ during the CMJ in both conditions do not confirm the previous suggestion of the co-existence of potentiation and fatigue based on the jumping F-t data after a running protocol therefore our initial hypothesis was rejected. Interestingly, the correlations found between changes in vertical stiffness and jumping height in the CMJ may suggest a change in jumping strategy after running exercises of different intensity, with and without the presence of peripheral fatigue.

Previous research reported enhanced jump capacity after different running exercises in endurance trained runners (9). For this reason, the evaluation of jump capacity in endurance runners to monitor fatigue has not been recommended, contrary to what is done with sprinters and team sport athletes (25). The post-running jump potentiation after incremental running protocols is an interesting phenomenon as only endurance trained runners can jump higher and hence exhibit and enhancement in muscle power just a few minutes after volitional exhaustion (10, 26, 27). Based on previous studies in the field, the expected co-existence of fatigue and potentiation was based on performance in jumping exercises (14, 28). Specifically, the reduction in F_peak_ at the end of the eccentric phase of the countermovement had been previously interpreted as a sign of the fatigue associated with exhaustion (14). The current results are contrary to this assumption as the runners exhibited a reduction in F_peak_ after both the incremental test and the submaximal running bout, while only a reduction in MVC and low-frequency fatigue were observed in the knee extensors after the incremental test. Further, the correlation between the changes in MVC and the ratio 10:100 would confirm the peripheral origin of this fatigue which may be associated to an excitation-contraction coupling failure. Therefore, the changes in the F-t curve during jumping cannot be interpreted as surrogates of muscle fatigue in this scenario. This is an important consideration that confirms the recent suggestions by Garcia-Pinillos et al. (25) of not using vertical jumps to evaluate running associated fatigue in endurance runners. In this regard, the loss of H_CMJ_ observed in 800-m runners during a 5 × 200 m session (29) may be debt to the combined effect of training background of athletes, the excessive peripheral fatigue associated to supramaximal short running bouts, and the time for CMJ evaluations which were performed just immediately after the end of every running bout. Future studies should verify if this assumption can be extended to other endurance athletes after different running exercises with the evaluation of CMJ at different time points during the first 10 min of recovery.

From a mechanistically point of view, the correlation found between changes in K_vert_ and H_CMJ_ may suggest that running before jumping may induce changes in the jumping strategy, independently of the running intensity and hence the levels of fatigue. Thus, the adjustments in the stiffness of the body system during the countermovement, with changes in both the force and the lowering of the center of mass, may be the biomechanical factors behind these changes in the F-t curve. These changes might be related to a different force-length relationship of the knee extensors and, thus, to a different recruitment strategy during the CMJ performed after both the EXP and CTR conditions. However, we cannot confirm this hypothesis with the current experiment, therefore further studies should test the jumping strategy to confirm or refute it with more appropriate methods. However, if these changes are associated to running-associated fatigue of other muscle groups (e.g., hamstrings, gastrocnemius) (30) or to changes in muscle activation patterns or elastic energy transfer (31) also requires further investigation. Meanwhile, the enhancement of jump capacity only after the incremental running test after exhaustion may be associated to PAP because of the greater work accumulated at submaximal intensities in the experimental condition (9). Finally, the association between jump potentiation and PAP evaluated with the TIT remains to be clarified in future studies.

The limitations of the current study include: (1) its low sample size which do not allow performance level or sex comparisons (32); (2) the absence of PAP evaluations to verify if PAPE and PAP actually co-exist (4) thus validating the use of CMJs as a surrogate of muscle potentiation in this context; (3) the absence of fatigue assessments of other muscles involved in jumping (30); (4) The applicability of the current findings to evaluations only performed a few minutes (<5 min) after incremental running tests until exhaustion; (5) The non-randomization of the CTR and EXP conditions performed in the same day. Therefore, future studies should elaborate on these factors to better understand the etiology of post-incremental running jump potentiation in endurance trained runners of different training backgrounds and competitive levels. Additionally, consideration of alternative jumping performance parameters would provide additional insights to a better understanding of the phenomena.

### Conclusions and practical applications

The results of the current brief research report confirm previous studies observing a post-incremental running jump potentiation in endurance trained runners. However, the decrement in maximum force during the CMJ observed in both the experimental (i.e., after the incremental running test after exhaustion) and the control (i.e., after a submaximal square-wave run) conditions may suggest that these changes in the F-t curve during jumping may not be indicative of the peripheral low-frequency fatigue confirmed with the TIT only in the experimental condition. Future studies are required to verify if the PAPE (i.e., jump potentiation) observed is also associated to PAP.

From a practical point of view, the current results confirm the recent suggestions that acute changes in jump performance may not be appropriate to evaluate acute fatigue after an incremental test until exhaustion in endurance trained runners as changes in jumping capacity may be also related to changes in jumping strategy and not only to knee extensors fatigue. CMJ performance in endurance athletes of different training backgrounds is a simple and useful monitoring tool to be used before and after different running exercises to monitor acute and chronic training adaptations (11, 33).

## Data Availability

The raw data supporting the conclusions of this article will be made available by the authors, without undue reservation.
