# Supplementary material for: Co-existence of peripheral fatigue of the knee extensors and jump potentiation after an incremental running test to exhaustion in endurance trained male runners
Source: Front Sports Act Living. 2023 Nov 9;5:1267593. doi: 10.3389/fspor.2023.1267593 (PMC10670794; doi:10.3389/fspor.2023.1267593)
Supplement: Supplementary file 1 [file Table1.docx]

| **Variable** | **ICC** | **CV** | **T.TEST** |
| --- | --- | --- | --- |
|  |  |  |  |
| **MVC** | 0,926 | 7,244 | 0,515 |
| **Db_100Hz_** | 0,958 | 5,042 | 0,694 |
| **Db_10Hz_** | 0,846 | 6,870 | 0,202 |
| **Tw_single_** | 0,926 | 4,578 | 0,661 |
| **Ratio 10:100** | 0,906 | 5,557 | 0,198 |
| **VA(%)** | 0,573 | 6,123 | 0,342 |
| **H_CMJ_** | 0,999 | 1,360 | 0,893 |
| **ΔL** | 0.832 | 8,788 | 0.809 |
| **F_peak_** | 0.877 | 4.839 | 0.652 |
| **K_leg_** | 0.582 | 10.607 | 0.381 |
| **PO_peak_** | 0.868 | 3.024 | 0.809 |

**Supplementary Table 1**. Reliability of selected neuromuscular variables.

*All the analyses were made comparing the PRE values of both conditions (CTR vs EXP). ICC: intraclass correlation coefficient; CV: coefficient of variation; T.TEST: student T test for paired comparisons.
